# Near Fatal Asthma: Clinical and Airway Biopsy Characteristics

**DOI:** 10.1155/2012/829608

**Published:** 2012-02-09

**Authors:** Richard G. Barbers, Ilias C. Papanikolaou, Michael N. Koss, Ashish Patel, Elton Katagihara, Maggie Arenas, Khalid Chan, Colleen G. Azen, Om P. Sharma

**Affiliations:** ^1^Division of Pulmonary and Critical Care Medicine, Department of Medicine, Keck School of Medicine, University of Southern California, Los Angeles, CA 90033, USA; ^2^Pulmonary Department, General Hospital of Corfu, Los Angeles, CA 90033, Corfu, Greece; ^3^Department of Pathology, University of Southern California, Los Angeles, CA 90033, USA; ^4^Department of Biostatistics, Clinical Trials Unit, University of Southern California, Los Angeles, CA 90033, USA

## Abstract

*Background*. Inflammation and remodeling are integral parts of asthma pathophysiology. We sought to describe the clinical and pathologic features of near fatal asthma exacerbation (NFE). *Methods*. Bronchial biopsies were collected prospectively from NFE I subjects. Another NFE II group and a moderate severity exacerbation control group (ME II) were retrospectively identified—no biopsies obtained. *Results*. All NFE II (*n* = 9) subjects exhibited remodeling and significant inflammation (eosinophilic, neutrophilic). NFE II group (*n* = 37) had a significant history of prior intubation and inhaled corticosteroids usage compared to ME II group (*n* = 41). They also exhibited leukocytosis, eosinophilia, and longer hospitalization days. *Conclusions*. Remodeling, eosinophilic, and neutrophilic inflammation were observed in NFE. NFE is associated with prior intubation and inhaled corticosteroids usage.

## 1. Introduction

Remodeling in asthma refers to structural changes in large and small airways, consisting of subepithelial fibrosis, increased vascularity, increased airway smooth muscle mass, and goblet cell hyperplasia of proximal and distal airways [1, 2]. Remodeling was believed originally to be the cause of refractory asthma, that is, asthma that fails to respond to optimal treatment and is characterized by persistent airflow limitation [3]. However, apart from severe asthma, bronchial biopsy studies have shown the features of remodeling in mild and moderate asthma, as well as in children with asthma and preschool wheezing [4, 5].

Remodeling and persistent inflammation are present in relatively mild and “benign” asthma, but not many data exist regarding the pathologic features in severe asthmatic exacerbations or in near fatal asthma [6]. This study was conducted in order to characterize the clinical and airway biopsy features of asthmatic patients who had a near fatal exacerbation (NFE). The NFE patients are severe asthmatics who presented at the Emergency Department (ED) requiring admission to the Intensive Care Unit (ICU) and were put under mechanical ventilation. We hypothesized that this subgroup of patients was the most likely to exhibit evidence of airway remodeling.

This observation would particularly help us how to optimally manage these severely compromised patients; studies in humans but also in animal models yield contradictory results about the effectiveness of anti-inflammatory medication against remodeling [7, 8]. The clinical phenotypes associated with an NFE are, as well, of obvious clinical importance.

## 2. Materials and Methods

### 2.1. Study Design

We defined two subgroups of asthma subjects who presented with an acute exacerbation at the ED. One group consisted of severe asthmatics with an NFE who required intubation and mechanical ventilation and were admitted to the ICU (NFE group). The second group consisted of asthma patients with a less severe exacerbation who were admitted either to the ICU or to a medical ward and preserved spontaneous breathing. The latter group responded to conventional therapy and was designated as the moderate exacerbation (ME) group (controls). The study comprised of a prospective and a retrospective arm.

### 2.2. Prospective Arm

Between 2004 and 2009, asthma patients admitted to Los Angeles County and University of Southern California (LAC and USC) Medical Center (a public university-affiliated hospital) and to the USC University Hospital (USCUH) (a private university-affiliated hospital) with an NFE were included prospectively in the study. Inclusion criteria were an NFE as defined previously and a diagnosis of asthma reported by the admitting and the ICU physician. Exclusion criteria were age below 16th year and history of Chronic Obstructive Pulmonary Disease (COPD). Patients underwent bronchoscopy and had mucosal biopsies of the large proximal airways. These patients comprised NFE I group. Unfortunately, no ME patients were prospectively included in the study; as a result, control biopsies were not available in this study.

### 2.3. Retrospective Arm

Because of the lack of a prospective control group, we retrieved in a retrospective fashion the medical records of adult asthmatics which presented at the ED and admitted to LAC and USC Medical Center or to USCUH between 1999 and 2004 with an asthma exacerbation. As defined previously, they were divided to those with an NFE (called NFE II group) and those with an ME (called ME II group). No biopsies were available for these retrospectively collected groups. The same inclusion and exclusion criteria with the prospective data were applied. The purpose of the retrospective arm was therefore to provide an analysis of NFE patients (having similar clinical and social characteristics with NFE I group) with a control ME group, unavailable in the prospective arm.

### 2.4. Baseline Characteristics

We reviewed the charts for recording of (i) medication used at time of admission (unfortunately doses of inhalers have not been recorded at the ED), (ii) smoking habit (in pack/years), and (iii) respiratory symptoms and signs, blood eosinophilia, and evidence of infection. Pulmonary function testing was not recorded in the charts. We believe that the explanation is twofold: the discomfort of the emergency setting at which admissions took place and the mainly low socioeconomic status of most patients which prevented them from regular and proper follow-up of their disease.

### 2.5. Institutional Review Board Approval

The study was approved by the University of Southern California Institutional Review Board (federal ID number: IRB1 00005904) for the medical chart review (retrospective group) as well as for the patients who underwent bronchoscopy and airway biopsies (prospective group). Informed consent was obtained from the patient directly or from the patient's legal surrogate.

### 2.6. Bronchoscopy

Bronchoscopy was performed through the endotracheal tube and mechanical ventilation was performed appropriately and according to the patient's best care and management. After each subject was stabilized with maximum conventional treatment and under sedation, bronchoscopy was performed at the bedside within 12 hours after intubation (using a Pentax bronchoscope). Five mucosal biopsies were taken from the right main bronchus, the bronchus intermedius, and from a lobar bronchus (middle lobe or upper lobe). The biopsies were superficial in order to prevent significant hemorrhage. Biopsy specimens were processed in the Pathology Department to be examined by one pathologist (MK).

### 2.7. Airway Biopsy Preparation and Processing

The airway tissues submitted in 10% formalin fixative were embedded in paraffin after appropriate dehydration and 5-micron-thick sections cut and stained as follows: one hematoxylin and eosin-stained slide, one PAS- (periodic acid-schiff-) stained slide, and one trichrome-stained slide with at least 2 sections per slide. The numbers of eosinophils, neutrophils, lymphocytes, and plasma cells in the highest area of cellularity of the bronchial wall were scored semiquantitatively as follows: 1–5 cells/HPF (high-power field) = 1+; 6–10 cells/HPF = 2+; >10 cells/HPF = 3+. Next, the respiratory basal lamina was graded as normal or thickened. The presence of mucinous and squamous metaplasia was determined by counting 30 contiguous respiratory epithelial cells and noting the percentage of mucinous or squamous cells among them. Bronchial wall edema was judged as mild, moderate, or severe. Finally, to determine the vascularity of the bronchial wall, the maximum number of capillaries in the airway wall was counted under a high-power microscopic field (×400).

### 2.8. Statistical Analysis

Values are presented as means ± standard deviation or frequency and percent. Measured values between groups NFE II and ME II were analyzed with two-tailed *t* test or a nonparametric equivalent (Mann-Whitney test). *χ* ^2^ or Fisher's exact test was performed for qualitative variables. Relative risk and its 95% confidence interval were calculated. Correlation analysis was performed in all groups. A Roc analysis in the retrospective arm was done to find the features with the largest area under the curve for subsequent intubation. Multiple logistic regression analysis was also performed to identify independent factors associated with intubation in the retrospective arm, although the large number of missing values for some key variables limited the utility of this approach. The threshold of statistical significance was *P* < 0.05.

## 3. Results

### 3.1. Prospective Arm

**Figure 1:**
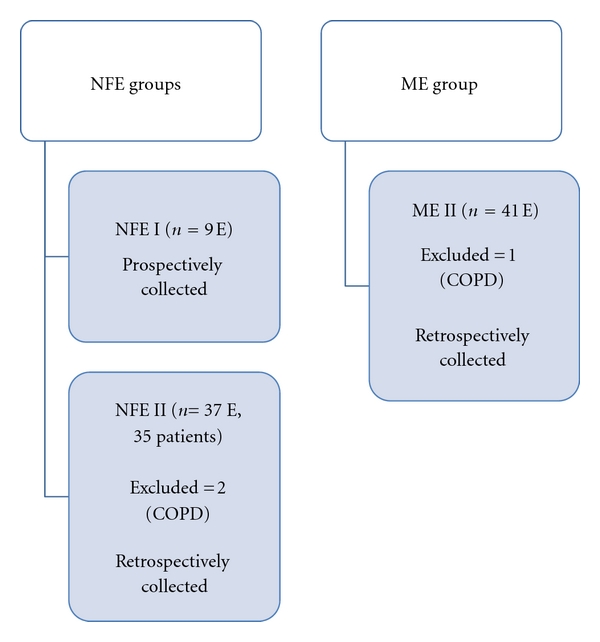
Flow diagram of study subjects and controls (NFE: near fatal exacerbation; ME: moderate exacerbation; E: exacerbation; COPD: chronic obstructive pulmonary disease).

Patients' classification in groups is shown in Figure 1. Patients' demographics are shown in Table 1 and their clinical characteristics in Table 2. Their history was remarkable for prior intubation for asthma exacerbation (67%) and recent use of systemic corticosteroids (89%). Only 1 patient (11%) was a smoker. Outcome was favorable for all 9 patients.

**Figure 2:**
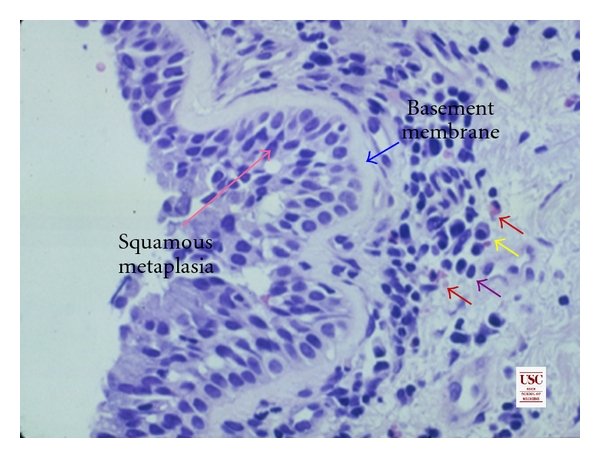
Light microscopic appearance of bronchial biopsy in asthma (magnification scale ×400). Features include early squamous metaplasia of bronchial epithelium, thickened respiratory epithelial basal lamina, and an underlying edematous bronchial wall showing prominent numbers of lymphocytes (purple arrow), some plasma cells (yellow arrow), and a few eosinophils (red arrows).

The histopathological findings of the NFE I subjects are shown in Table 3. All specimens exhibited increased vascularity, basal lamina thickening, mild to moderate edema, significant inflammation, as well as various degrees of mucinous and squamous metaplasia (Figure 2). No airway smooth muscle cells were retrieved in the specimens, probably because of the superficially obtained biopsies. Eosinophils and lymphocytes were the predominant cells found. Neutrophils and plasma cells were also increased and, in some cases, equivalent to the eosinophil population. There was no significant correlation of the biopsy results with the clinical features or laboratory results.

### 3.2. Retrospective Arm Analysis

The retrospective arm consisted of groups NFE II and ME II. 2 patients had 2 severe exacerbations each. Each exacerbation was considered a separate event (Figure 1). Patients' demographics are shown in Table 1. There were missing data for most variables. Clinical characteristics of groups NFE II and ME II are shown in Table 4. No deaths were recorded in these 2 groups.

The NFE II group exhibited higher heart rate (HR), respiratory rate (RR), and diastolic blood pressure (DBP). They had higher FiO_2_ administered, higher arterial PO_2_ and PCO_2_, and lower pH than the ME II group. They also had profound leukocytosis, higher peripheral blood eosinophils (percentage and absolute counts), as well as longer hospitalization days (Mann-Whitney test, *P* < 0.05). In addition, these patients exhibited higher alveolar-arterial oxygen gradient (A-aO_2_) but did not reach statistical significance (*P* = 0.09). Upper respiratory tract infection was common but not different between study subjects and controls.

NFE II group had a significant history of prior intubation and prior usage of inhaled corticosteroids comparing to controls (chi-square test, *P* < 0.05). Doses of corticosteroids have not been sufficiently recorded into the charts and are missing from our study. Multiple logistic regression conducted with covariates that had no more that 25% missing data identified DBP (*P* = 0.0041) and RR (*P* = 0.0024) as independent clinical risk factors for intubation. The Roc analysis determined that the length of hospitalization, absolute eosinophils, and per cent eosinophilia had the larger area under the curve for intubation (0.89, 0.85, 0.84, resp.).

## 4. Discussion

The main outcome and the primary hypothesis of the present study is that, in near fatal asthma exacerbators, remodeling of the proximal airways along with cellular inflammation was commonly observed. To our knowledge, this is the second study of bronchial biopsies performed in living subjects with a severe asthma exacerbation, even in a semiquantitative manner. Although these NFE I patients had a significant history of prior intubation due to asthma and usage of inhaled and systemically administered corticosteroids, association of this phenotype with the specific pathology findings cannot be clearly made due to the lack of a biopsied control ME I group.

Retrospective NFE II subjects in which biopsies were not performed exhibited leukocytosis, peripheral blood eosinophilia, and prolonged hospitalization. Independent clinical risk factors for intubation were higher diastolic blood pressure and greater respiratory rate; however, these results should be interpreted with caution due to missing data. These subjects had a significant history of prior intubation and use of inhaled corticosteroids compared to controls.

The major limitation of our study is the lack of a biopsied control group (moderate asthma exacerbations). Although they may also exhibit remodeling, our primary goal was to investigate the pathology of the most severe asthma population. No conclusions can be made regarding the clinical characteristics associated with remodeling in those cases, which was the secondary purpose of this study. Retrospectively collected NFE II group exhibited, however, very similar characteristics with NFE I group in terms of age, race, sex, history, and medication used. They also had a similar mainly low socioeconomic status as most of them were underinsured and the study derived from a public hospital. As a result, we believe that clinical characteristics of NFE II patients, which are compatible with the currently known phenotype of Near Fatal Asthma (NFA) (frequent ED visits, prior intubation), may not be extrapolated to NFE I patients and therefore associated with remodeling. Retrospective population provides, however, a very good reflection of the prospective arm of the study.

A second limitation is the lack of pulmonary function testing and of other missing data (detailed smoking history, doses of medication). We attribute the former to the poor monitoring of asthma that these patients showed, an element explained in detail here in after. Regarding the latter, these data have been collected retrospectively from charts frequently over a decade old and were unable to be retrieved. A third limitation of our study is the only semiquantitative description of tissue specimens. We acknowledge that limitation and hope to be addressed in a future study.

Remodeling in near fatal asthma is a reasonable finding, along with evidence of acute and chronic inflammation. Epithelial basal lamina thickening, increased smooth muscle mass, vascularity, and goblet cell hyperplasia are all observed in various asthma stages (mild, moderate, and severe) [9–12]. Although all of our patients survived, its impact on short term prognosis of an exacerbation was not addressed in this study and remains to be elucidated.

A major finding in the tissue specimens of near fatal asthmatics in our study is the combination of predominant eosinophilic inflammation with remodeling. While eosinophils have been associated, as found in our study, with persistent, severe asthma, their role in the pathogenesis of remodeling is not so clear. Eosinophils have been shown to induce remodeling through the production of a strong fibrogenic cytokine, TGF-*β* (transforming growth factor-*β*), conversion of fibroblasts to myofibroblasts, and excessive extracellular matrix expression (ECM). Phipps et al. and Flood-Page and colleagues found that patients with atopic asthma who received mepolizumab, an anti-IL5 antibody, exhibited a decrease in lung eosinophilia along with a reduction of markers of remodeling, suggesting a close association [13, 14]. Mice models have shown, however, that remodeling persists after resolution of the inflammation [15, 16]. Unfortunately, subsequent biopsies were not obtained after asthmatic crisis resolution in our study.

The use of inhaled corticosteroids (CSs) was rather low in our study subjects (17% in ME II group and 48% in NFE II group) and signifies nonadherence to current treatment guidelines [17]. This underlines, to our opinion, the undertreatment of asthma in the majority of our patients, due to socioeconomic status and underinsurance. This undertreatment may be the cause of the peripheral eosinophilia noticed in the retrospective data. This study could not address the issue of the effect of CS on remodeling in NFE biopsied group. Other studies have yielded contradictory results [18, 19]. Thermoplasty and novel targeted therapies may prove much more beneficial against remodeling [20, 21]. Another feature of NFE II asthmatics was peripheral blood leukocytosis. Possible explanations for the leukocytosis might be the use of systemic corticosteroids or an upper respiratory tract infection (URTI).

Although cases of COPD have been excluded from the study, a significant proportion of study subjects were current or former smokers. Thus, certain of the study subjects might have coexistent asthma and COPD or may have been misdiagnosed as asthma instead of COPD. In the absence of pulmonary function testing and scheduled posthospitalization visit, this issue may not be addressed adequately.

## 5. Conclusion

In conclusion, this is a descriptive study showing that eosinophilic inflammation and remodeling are the predominant features of near fatal asthma exacerbations. These exacerbations are associated with a history of prior endotracheal intubation and use of inhaled CS. The lack of a moderate severity exacerbation control group, pulmonary function testing, and the semiquantitative pathological description is the limitations of our study.

## Figures and Tables

**Table 1:** Patients' demographics. Data are presented as frequency (percent) or as mean ± standard deviation (*n*).

	NFE I group	ME II group*	NFE II group*
Males	4/9 = 44%	20/41 = 48.8%	17/35 = 48%
Age (years)	48.5 ± 15 (9)	48 ± 15 (34)	50 ± 16 (24)
Race	*n* = 9	*n* = 39	*n* = 34
African-American	1 (11.1%)	14 (35.9%)	7 (20.6%)
Caucasian	2 (22.2%)	6 (15.4%)	4 (11.8%)
Hispanic	5 (55.6%)	14 (35.9%)	16 (47%)
Asian	1 (11.1%)	5 (12.8%)	7 (20.6%)
Smoking history	1/9 (11%)	12/28 (43%)	8/26 (31%)
Pack/years		10 ± 3.5 (4)	8.5 ± 6 (3)
Years with asthma	21.5 ± 9 (9)	17 ± 14 (21)	13 ± 9.5 (23)

*P values between NFE II and ME II groups for age, sex, race, smoking habit, pack/years of smoking, and years with asthma: nonsignificant.

**Table 2:** Clinical characteristics of NFE I group at the initial evaluation. Values are shown as mean ± standard deviation or frequency and percent.

	NFE I (*n* = 9)
Prior admissions	7 (78%)
Prior intubation	6 (67%)
B_2_ agonist use	9 (100%)
Inhaled corticosteroids	8 (89%)
Systemic corticosteroids the previous month	8 (89%)
Heart rate (beats/min)	126 ± 7
Respiratory rate (breaths/min)	30 ± 6
Systolic BP (mmHg)	124 ± 21
Diastolic BP (mmHg)	66 ± 20
FiO_2 _(%)	59 ± 30
PO_2_ (mmHg)	201 ± 137
PCO_2_ (mmHg)	42 ± 16
pH	7.3 ± 0.12
A-aO_2_ (mmHg)	186 ± 119
WBC (mm^3^)	16,286 ± 5,580
Eos (%)	0.33 ± 0.26
Eos absolute (mm^3^)	54 ± 50
BUN (mg/dl)	30 ± 27
Hospitalization days	7.3 ± 4.8

BP: blood pressure; WBC: white blood counts; Eos: eosinophils; A-a: alveolar-arterial gradient; BUN: blood urea nitrogen.

**Table 3:** Pathology findings of large airways in near fatal asthma exacerbation (NFE I group, *n* = 9).

Subject	1	2	3	4	5	6	7	8	9
Eosinophils	3+	3+	3+	3+	0+	1+	0+	3+	1+
Neutrophils	1+	1+	1+	3+	1+	1+	1+	3+	1+
Lymphocytes	3+	3+	3+	3+	2+	2+	2+	3+	3+
Plasma cells	1+	2+	2+	3+	1+	1+	2+	2+	1+
Mucinous metaplasia	0%	30%	15%	75%	15%	15%	30%	75%	30%
Squamous metaplasia	100%	20%	30%	50%	10%	20%	30%	20%	50%
Inflammation	3+	3+	2+	3+	1+	2+	3+	3+	3+
Basal lamina	thick	thick	thick	thick	thick	thick	thick	thick	thick
Edema	mild	moderate	moderate	moderate	mild	moderate	moderate	moderate	mild
Vascularity Vessels/hpf	10+	10+	10+	10+	10+	10+	10+	9+	9+

**Table 4:** Clinical characteristics of NFE II and ME II groups at the initial evaluation. Values are shown as mean ± standard deviation (*n*) or frequency and percent. The relative risk is presented at the last column.

	ME II	NFE II	*P* Value	RR (95% confidence interval)
Prior admissions	19/23 (83%)	22/25 (88%)	NS	
Prior intubation	9/30 (30%)	16/27 (59%)	0.03	1.97 (1–3.7)
B_2_ agonist Use	23/25 (92%)	30/33 (91%)	NS	
Inhaled corticosteroids	6/36 (17%)	16/33 (48%)	0.008	2.9 (1.2–6.5)
Systemic corticosteroids	11/36 (30%)	10/33 (30%)	NS	
Cough	10/12 (83%)	15/20 (75%)	NS	
Wheezing	10/12 (83%)	22/23 (96%)	NS	
Sputum	6/10 (60%)	13/17 (76%)	NS	
Chest tightness	3/6 (50%)	6/10 (60%)	NS	
URTI	6/16 (37%)	7/15 (47%)	NS	
Heart rate (beats/min)	102 ± 21 (38)	124 ± 20 (35)	0.0001	
Respiratory rate (breaths/min)	25 ± 6 (38)	32 ± 7 (25)	<0.0001	
Systolic BP (mmHg)	136 ± 19 (38)	147 ± 39 (36)	NS	
Diastolic BP (mmHg)	75 ± 18 (37)	93 ± 25 (34)	0.002	
PO_2_ (mmHg)	85 ± 33 (25)	209 ± 170 (34)	0.003	
PCO_2_ (mmHg)	41 ± 10 (25)	57 ± 18 (34)	0.001	
pH	7.38 ± 0.06 (24)	7.27 ± 0.12 (34)	0.0001	
FiO_2_ (%)	40 ± 21 (19)	71 ± 32 (26)	0.001	
A-aO_2_ (mmHg)	136 ± 128 (19)	188 ± 142 (26)	0.09	
WBC (mm^3^)	11,500 ± 4,000 (29)	15,600 ± 6,200 (25)	0.01	
Eos (%)	1 ± 1.4 (19)	2.75 ± 1.5 (4)	0.03	
Eos absolute (/mm^3^)	125 ± 198 (19)	445 ± 324 (4)	0.03	
Hospitalization days	3.8 ± 2.6 (27)	9.8 ± 5 (7)	0.001	

URTI: upper respiratory tract infection; BP: blood pressure; WBC: white blood counts; Eos: eosinophils; A-a: alveolar-arterial gradient; NS: nonsignificant.

## Abbreviation List

DBP: Diastolic blood pressure
ECM: Extra cellular matrix
HR: Heart rate
ICU: Intensive care unit
ME: Moderate exacerbation
NFA: Near fatal asthma
NFE: Near fatal exacerbation
RR: Respiratory rate.
